# Epidemiology of Gastrointestinal Nematodes in Extensively Managed Pastoral Small Ruminants: The Case of Nyangatom District, South Omo Zone, Southern Ethiopia

**DOI:** 10.1155/japr/6971937

**Published:** 2026-06-17

**Authors:** Yebelayhun Mulugeta, Getachew Mulugeta, Nahom Belay, Mahider Tesfaye

**Affiliations:** ^1^ College of Veterinary Medicine and Animal Sciences, Jinka University, Jinka, Ethiopia; ^2^ Nyangatom District Livestock and Fishery Development Office, Kangaten, Ethiopia; ^3^ Bonga Agricultural Research Center, Bonga, Ethiopia

**Keywords:** gastrointestinal, nematodes, Nyangatom, prevalence, risk factors, small ruminants

## Abstract

Gastrointestinal nematode infections pose a significant challenge to the productivity and reproductive performance of small ruminants in Ethiopia. This study is aimed at determining the prevalence of gastrointestinal nematode parasites in small ruminants in one of the pastoral areas of Ethiopia, identifying the main gastrointestinal tract (GIT) parasites, and assessing potential risk factors linked to their occurrence. A cross‐sectional study was conducted from June to November 2025 to determine the prevalence of gastrointestinal nematodes in sheep and goats in Nyangatom District, Southern Ethiopia, using both qualitative and quantitative coprological examinations. A total of 384 small ruminants, 176 sheep and 208 goats, were systematically selected from the study population and examined for GIT nematode infection. Of these, 253 (65.89%) tested positive for one or more GIT nematodes. The prevalence was 71.59% in sheep and 61.06% in goats. Among the samples, 198 (51.56%) were positive for *strongyle* eggs, 15 (3.91%) for *Strongyloides* eggs, 9 (2.34%) for *Trichuris* spp. eggs, and 31 (8.07%) for mixed infections. Three risk factors, namely study kebeles, species, and body condition score (BCS), were statistically linked to GIT nematode prevalence. These results highlight the significant impact of gastrointestinal nematode infections on small ruminant health and productivity in the study area. The high prevalence rates call for urgent implementation of effective control measures. Strategic deworming programs should be adopted to reduce infection burdens, and animal health extension workers need to actively educate local communities about parasite control. Overall, this study emphasizes the necessity of integrated parasite management strategies to reduce the negative effects of gastrointestinal nematodes.

## 1. Introduction

Ethiopia owns one of the largest ruminant populations, comprising more than 60.39 million heads of cattle, 42.91 million sheep, and 52.46 million goats [1]. Due to their hardiness and fecundity, small ruminants play a crucial role in the cultural and socioeconomic lives of rural people in Africa, providing meat, milk, income, and wealth. However, the productivity of small ruminants in the country is limited by widespread health problems, poor nutrition, poor genetic quality of indigenous stock, poor husbandry practices, and limited technological support [2].

Gastrointestinal nematode (GIN) infections are among the leading diseases impacting the productive and reproductive performance of small ruminants in Ethiopia [3]. Gastrointestinal parasites are responsible for serious economic losses to sheep and goat‐producing enterprises through heightened susceptibility of animals to other infections, morbidity, and mortality, especially in densely parasitized animals and in newborn and growing animals. Not to mention, they expedite enforced culling, meat and organ condemnations, heightened cost of veterinary therapy, decreased weight gain, reduced milk production and reproductive magnitude, reduced work scope, decreased food intake, decreased animal growth rates, and lessened weight gains and treatment and management expenditures ([4, 5]; and [6]).

Various risk factors play a crucial role in the onset of GIN infections, influenced by both host and environmental factors. Environmental factors include agro‐ecological conditions and animal husbandry practices, such as housing systems, deworming schedules, and pasture management [7]. These factors primarily determine the type, incidence, and severity of numerous parasitic infections [8]. Additional risk factors, such as host species, sex, age, body condition score (BCS), and breed [9], as well as parasite species and worm population density, influence the development of gastrointestinal parasitic infections [10].

Numerous studies have been conducted to assess the prevalence of nematode parasites across all small ruminant‐rearing regions in Ethiopia ([11–13]; and [14]). GINs are highly prevalent among small ruminants in pastoral areas of Ethiopia, posing a significant challenge to animal health and productivity. National‐level studies and numerous regional surveys consistently report high infection rates, with pooled prevalence estimates around 75%–80% for sheep and goats, though local rates can range from about 50% to over 90% depending on the area and management practices [15–20]. The most commonly identified nematode genera include Haemonchus, Trichostrongylus, Teladorsagia/Ostertagia, Strongyloides, Oesophagostomum, and Trichuris [15–20].

Despite the substantial population of shoats in the current pastoral area, gastrointestinal tract (GIT) nematodes, although a chronic and production‐limiting major health concern in pastoral systems, remain unexplored, with limited data available on their prevalence and associated risk factors. Information on these aspects would be instrumental in devising control strategies and preventing nematode infections. Consequently, the primary objectives of this study were to estimate the prevalence of GIN parasitism in small ruminants in an extensively managed pastoral area, identify the major gastrointestinal parasite species, and evaluate the risk factors associated with its incidence. Furthermore, the study is aimed at determining the significance of parasite infections and proposing strategic preventive and control measures to enhance the productivity of this sector.

## 2. Materials and Methods

### 2.1. Study Area

**Figure 1:**
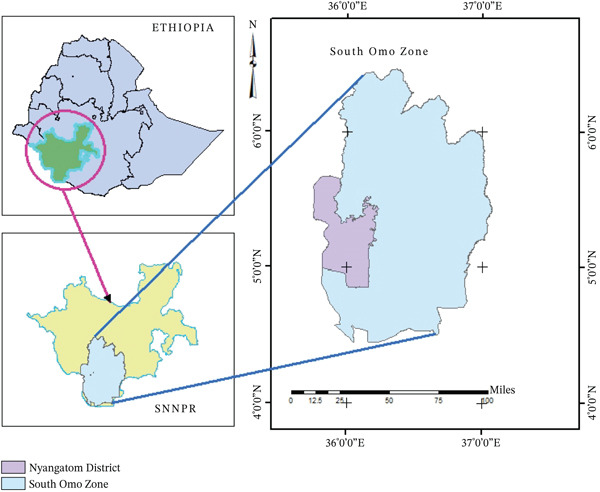
Location of the study area on the map.

The present study was conducted in the Nyangatom District, Southern Ethiopia (Figure 1). Nyangatom District is situated in the South Omo Zone of Southern Ethiopia, including 20 (1 urban and 19 pastoral) kebele administrations (KAs). It is among the seven districts in South Omo Zone with an area of 2652 km^2^ and is positioned at 4.85°–5.67° N and 35.75°–36.23° E. It is bordered by the West Omo Zone and Selamago District in the north, Dassenech District in the South, Hamar District in the east, and Kenya and South Sudan in the west. The agro‐ecology of the district is lowland (“kola”) with an altitude that ranges between 300 and 450 m.a.s.l. The mean annual temperature of the district ranges from 33°C to 42°C. The district has a rainfall type of bimodal type (“Belg” from March to May and “Meher” from August to October). The rainfall pattern in the district is erratic in nature. The mean annual rainfall ranges from 350 to 500 mm. Livestock production is the dominant farming system in the district. It has an animal population of approximately 520,586 cattle, 178,385 goats, and 136,940 sheep [1].

### 2.2. Study Design

A cross‐sectional study was conducted from June 2024 to November 2025 to estimate the prevalence of sheep and goat GIT nematodes by qualitative and quantitative coprological examination. All animals were deliberately identified, and data like sex, age, species, and BCS were registered. These risk factors were evaluated for the existence of a possible association with the presence of GIT nematodes.

### 2.3. Study Animals

The study animals were indigenous breeds of small ruminants maintained under an extensive management system. An ample number of sheep and goats are kept in the study area. Animals were sampled from approximately 48 pastoral households, with flock sizes ranging from 5 to 15 animals per household, and are used primarily for income generation and meat production. Similarly, in many parts of the country, the feed source for sheep and goats is natural grazing land and crop residues, with absolutely no extra supplement and minimal health intervention. At the time of sampling, data concerning age, sex, fecal consistency, body condition, and other important risk factors were collected for individually sampled animals. The age of the animals was identified based on the farmers′ feedback and teeth checkup. Animals were categorized into three age groups for both sheep and goats as follows: young (< 1 year), adult (1–3 years), and old (> 3 years). BCS was determined following Morgan et al. [21] report as 1 (*poor*), 2 (*average or medium*), or 3 (*good*).

### 2.4. Sampling Methods

Animals were eligible for inclusion if they were older than 3 months and had not received anthelmintic treatment within 1 month prior to sampling. The sample size necessary for the study was calculated by using the formula forwarded by Thrusfield et al. [22] for a simple random sampling technique. A 5% absolute precision and 95% confidence interval were considered for the sake of calculating sample size, considering an expected prevalence of 50%.

n=1.962×Pexp×1−Pexpd2

Subsequently, a total of 384 small ruminants, including 176 sheep and 208 goats, were selected systematically from the study population and investigated for GI nematode infection. Fecal samples were collected directly from the rectum of the study animals using sterile gloves and stored in plastic, labeled fecal cups. The samples were then transported to the laboratory in an ice box on the same day of collection and later stored at 4°C until coprological examination.

### 2.5. Study Methodology

In this study, approximately 10 g of fecal sample was collected directly from the rectum of each animal using disposable gloves and placed into a clean, labeled plastic universal bottle. To preserve parasite egg morphology and prevent further development, 10% formalin was added to each sample. Samples were transported in an ice box to the laboratory on the day of collection and stored at 4°C until examination. Qualitative coprological examination was performed using sedimentation and flotation techniques. For flotation, a saturated sodium chloride solution with a specific gravity of approximately 1.20 was used. About 3 g of faces was mixed with flotation solution, strained, and transferred into a test tube. The tube was filled to form a convex meniscus, covered with a coverslip, and allowed to stand for 10 min before microscopic examination.

Sedimentation was performed by emulsifying fecal material in water, straining the mixture, and allowing it to settle. The supernatant was discarded, and the sediment was examined microscopically for heavier parasite eggs. Quantitative examination was carried out using the modified McMaster egg counting technique. Fecal egg counts (FECs) were expressed as eggs per gram (EPG) of feces following standard procedures [23]. Identification of GINs was conducted at the genus level based on egg morphology, size, and structural characteristics using standard parasitological keys [24], as molecular diagnostic methods were not employed. The intensity of infection was classified as light (50–800 EPG), moderate (800–1200 EPG), and heavy (> 1200 EPG) infections [25].

### 2.6. Data Management and Analysis

Data collected from individual animals and laboratory examinations were coded and entered into Microsoft Excel 2019 and analyzed using Stata Version 27. Risk factors for GIN infection were conceptually grouped into two hierarchical levels: (i) area‐/management‐level factors and (ii) individual animal‐level factors. Area‐level factors included study kebele (location), which reflects shared environmental conditions, grazing systems, and general management practices within each pastoral area. Individual animal‐level factors included species (sheep or goat), sex, age category, fecal consistency, and BCS. Univariable logistic regression analysis was first performed separately for each explanatory variable to assess its association with GIN infection. Variables with a *p* value < 0.05 were considered for further analysis. Subsequently, multivariable logistic regression analysis was conducted by including both area‐level and individual animal‐level factors in the same model to evaluate their independent effects while controlling for potential confounding. The hierarchical nature of the variables was considered during interpretation, recognizing that animals within the same kebele may share common environmental and management‐related exposures. Statistical significance was determined at a 95% confidence level, and associations with *p* < 0.05 were considered statistically significant.

## 3. Result

### 3.1. Prevalence

Out of the 384 small ruminants examined, 253 animals were positive for one or more GINs, giving an overall prevalence of 65.89% (95% CI: 61.0–70.6). When stratified by species, species‐specific prevalence was 71.59% (95% CI: 64.5–78.0) in sheep and 61.06% (95% CI: 54.2–67.6) in goats. Based on coprological identification, *strongyle* spp. were the most frequently detected parasites (51.56%), followed by *Strongyloides* spp. (3.91%), *Trichuris* spp. (2.34%), and mixed infections (8.07%), where more than one nematode genus was detected in the same animal (Table 1).

**Table 1:** Prevalence of gastrointestinal nematode genera in small ruminants (*N* = 384).

Species	No. positive	Prevalence (%)
*Strongyle* spp.	198	51.56
*Strongyloides* spp.	15	3.91
*Trichuris* spp.	9	2.34
Mixed infections	31	8.07
Total	253	65.89

Mixed infections refer to the concurrent presence of two or more nematode genera, primarily combinations of *strongyle* spp. with *Strongyloides* spp. or *Trichuris* spp. Mixed infections were observed in 8.07% of the examined animals. Such co‐infections may exacerbate parasitic burden, increase pasture contamination, and complicate control strategies. Although species‐level identification was not performed, some nematodes within these genera have recognized zoonotic potential, highlighting possible public health implications, particularly in pastoral communities with close human–animal interaction.

From the study areas, high prevalence of GINs was observed in Kangaten (74.19%) followed by Kopiriya (74.04%) (Table 2).

**Table 2:** Prevalence of GIT nematodes by study areas (*N* = 384).

Study kebeles	No. examined	No. positive	Prevalence (%)
Napsmury	101	51	50.50
Aipa	117	79	67.52
Kangaten	62	46	74.19
Kopiriya	104	77	74.04
Total	384	253	65.89

Fecal samples positive for GIT nematodes in this study were subjected to a McMaster egg counting chamber for EPG count to determine the intensity of infection. The majority of positive study animals had the EPG count in an average of less than 800. As to the level of infection, 12.62%, 25.69%, and 61.66% of sheep and goats were observed to be severely, moderately, and lightly infected, respectively (Table 3).

**Table 3:** Intensity of gastrointestinal nematode infection among infected animals (*N* = 253).

Level of infection	No. infected	Frequency	Prevalence
Light	253	156	61.66
Moderate	253	65	25.69
Severe	253	32	12.62
Total	253	253	100.00

### 3.2. Risk Factors

In the present study, factors like study areas, species, sex, age, fecal consistency, and BCS were supposed to have an impact on the prevalence of GI nematodes. These risk factors were evaluated at two levels. Study kebele was considered an area‐level factor representing shared environmental and management conditions, whereas species, sex, age category, fecal consistency, and BCS were considered individual animal‐level factors. Unless otherwise specified, risk factor analyses were conducted on pooled data from both sheep and goats; however, species was retained as an independent explanatory variable in both univariable and multivariable models.

Hence, univariate logistic regression analysis was used to observe the possible statistical association between the prevalence of the nematodes and the hypothesized risk factors. Accordingly, study areas, species, fecal consistency, and BCS were found to have statistically significant associations (*p* < 0.05) with the prevalence of GI nematodes in the study districts (Table 4).

**Table 4:** Univariate logistic regression results on different risk factors.

Risk factors	No. examined	No. positive	Prevalence (%)	OR	CI (95%)	*p* value
Study area						
Napsmury	101	51	50.50	Ref.		
Aipa	117	79	67.52	2.04	1.18–3.53	0.011 ^∗^
Kangaten	62	46	74.19	2.82	1.41–5.62	0.003 ^∗^
Kopiriya	104	77	74.04	2.80	1.55–5.03	0.001 ^∗^
Species						
Caprine	208	127	61.06	Ref.		
Ovine	176	126	71.59	1.61	1.05–2.47	0.031 ^∗^
Sex						
Male	103	70	67.96	Ref.		
Female	281	183	65.12	0.88	0.54–1.42	0.604
Age						
Young	109	76	69.72	Ref.		
Adult	181	118	65.19	0.81	0.49–1.35	0.427
Old	94	59	62.77	0.73	0.41–1.31	0.296
Fecal consistency						
Normal	205	135	65.85	Ref.		
Soft	102	72	70.59	1.24	0.74–2.08	0.405
Hard	77	46	59.74	0.77	0.45–1.32	0.341
Body condition score						
Good	95	47	49.47	Ref.		
Medium	143	97	67.83	2.15	1.26–3.67	0.005 ^∗^
Poor	146	109	74.66	3.01	1.74–5.21	0.001 ^∗^

CI, confidence interval; no., number; OR, odds ratio.

^∗^ *p* < 0.05.

The study kebele showed a significant association with GIN prevalence, indicating the influence of area‐level environmental and management conditions. Individual animal‐level factors, particularly species and BCS, also showed significant associations, demonstrating that infection risk is influenced by both shared area‐level exposures and individual host characteristics.

In the multivariable logistic regression analysis, variables were further subjected to backward stepwise multivariable logistic regression analysis. Accordingly, the final model showed that the three factors (study areas, species, and BCS) have a statistical association with the prevalence of GI nematodes in the study districts (Table 5).

**Table 5:** Multivariable logistic regression results on different risk factors.

Risk factors	No. examined	No. positive	Prevalence (%)	OR	CI (95%)	*p* value
Study area						
Napsmury	101	51	50.50	Ref.		
Aipa	117	79	67.52	2.89	1.49–5.60	0.002
Kangaten	62	46	74.19	4.19	1.92–9.15	0.001
Kopiriya	104	77	74.04	4.63	2.31–9.31	0.001
Species						
Caprine	208	127	61.06	Ref.		
Ovine	176	126	71.59	2.74	1.59–4.71	0.001
Body condition score						
Good	95	47	49.47	Ref.		
Medium	143	97	67.83	2.02	1.14–3.59	0.016
Poor	146	109	74.66	3.11	1.73–5.60	0.001
Constant				0.15	0.04–0.60	0.008

CI, confidence interval; no., number; OR, odds ratio.

## 4. Discussion

The present study revealed the presence of GIT nematode parasites with an overall prevalence of 65.89% in sheep and goats. The relatively high prevalence observed in this study may be associated with prevailing management practices in the study area, including communal grazing, limited parasite control measures, and increased environmental contamination with infective larvae. Similar patterns have been reported in previous studies conducted under traditional production systems [26]. The present result is consistent with previous reports by Dilgasa et al. [27,28]. The current result was higher than the reports of Abebe et al. [29], Ahmed et al. [30], Elemo and Geresu [31], Getachew et al. [32], Mohammed et al. [33], and Muluneh et al. [34]. However, the observed prevalence was much lower than previous studies [14, 35, 36]. The differences in prevalence observed between current and previous studies may be attributed to variations in geographical and climatic factors. In Ethiopia, where extreme temperatures and rainfall patterns occur, altitude plays a significant role. These elements could greatly impact the development, distribution, and survival of nematode parasites [37].

In the study area, livestock production is characterized by extensive management systems, where animals are frequently exposed to communal grazing lands. Limited veterinary supervision, absence of structured deworming programs, and inadequate nutritional support may contribute to sustained parasite transmission and reinfection cycles. Information gathered from livestock owners and local animal health professionals revealed that deworming practices were inconsistent, lacking strategic planning, and often reliant on the availability of drugs rather than being informed by epidemiological evidence.

Higher prevalence of GIT parasites was observed in sheep than in goats which is consistent with previous reports of Abebe et al. [29], Ahmed et al. [30], Dugassa et al. [38], Mohammed et al. [33], and Getachew et al. [32]. However, it is in contrary with other reports in Ethiopia where high prevalence was observed in goats than sheep [27, 28, 31, 34]. The high prevalence of infections in sheep may be linked to their grazing behavior. Sheep typically graze close to the ground, making them more susceptible to exposure to infective larvae. In contrast, free‐ranging goats tend to browse at higher levels, which reduces their contact with these larvae, resulting in lower exposure [39].

In this study, infections with *strongyles* were the dominant among species of nematodes in the small ruminants. Infections with *Strongyloides* spp. and *Trichuris* were also identified with minimum proportions. This is in agreement with several studies [27, 28, 31, 32, 40] who reported a high proportion of *strongyle* infection. This might be because ruminants have different levels of resistance to different species of parasitic infections [24].

Among the potential risk factors considered for this study, the study area, species, and BCS were significantly associated (*p* < 0.05) with GIN infection in small ruminants. Significant difference in prevalence was observed between sheep and goats, sheep having a higher prevalence. This result is in accordance with other reports that considered species as a potential risk factor [33, 40, 41].

In the present study, significantly higher prevalence of GIN infection was documented in small ruminants with poor BCSs (OR = 3.11, *p* < 0.05). This finding is in agreement with the reports of Abebe et al. [29], Elemo and Geresu [31], Getachew et al. [32], and Dugassa et al. [38]. This is because clinically, GIN infections are characterized by loss of appetite and diarrhea, and among the ramifications of these two clinical problems is loss of body condition [24].

However, there was no statistically significant difference in GIT parasite infections between the sex groups of animals (*p* > 0.05) in the current study. This finding was in agreement with the reports of Muluneh et al. [34], [27, 28]), Mohammed et al. [33], Getachew et al. [32], and Dugassa et al. [38], which showed that there was no statistically significant difference between sex groups in association with the prevalence of GIT parasites in small ruminants. The similarity of the degree of infection among the sex groups of animals in the present study might be due to both sexes of animals were exposed to a similar management system. However, this observation disagrees with the work of Negasi et al. [42], Sheferaw et al. [43], and Elemo and Geresu [31], who reported a higher prevalence of GIT nematodes in females than in males. It is assumed that females are more vulnerable to parasitic infections, especially in the course of pregnancy and periparturient stage, due to both stress and decreased immune status [44].

The current study′s age‐wise analysis indicated no statistically significant differences in GIT parasite infections across various age groups. This aligns with the findings of Muluneh et al. [34], Mohammed et al. [33], Getachew et al. [32], and Dugassa et al. [38], which also reported similar results, suggesting that GIT parasites do not significantly discriminate among age groups. In contrast, our findings contradict those from other studies conducted in different regions of Ethiopia [31, 40, 41], which indicated that younger animals are more susceptible to parasitic infections compared to their older counterparts. Furthermore, variations in infection prevalence among different species and body condition categories may be influenced by differences in immune competence. Animals in poor body condition tend to be immunocompromised, making them more vulnerable to parasitic infections. Additionally, physiological stressors such as pregnancy and lactation can further weaken immune responses, particularly in female animals.

It is important to note that the identified risk factors operate at different hierarchical levels. The association observed with study kebele reflects area‐level influences, such as communal grazing practices, climatic conditions, and general herd health management that affect all animals within a location. In contrast, factors such as species and BCS represent individual animal‐level susceptibility, which may influence exposure, immune competence, and infection outcome. Although clustering at the flock level was not explicitly modeled, this distinction was considered during interpretation of the results.

Mixed GIN infections may intensify clinical impact through synergistic effects, leading to increased production losses. Moreover, environmental contamination associated with heavy parasitic loads may pose indirect risks to human health, especially in pastoral systems where close contact between livestock and humans is common.

## 5. Limitations

Although FAMACHA scoring is a valuable field tool for assessing anemia associated with haemonchosis, it was not included in the present study. This represents a limitation and should be incorporated in future studies. Another limitation of this study is that detailed flock‐level management variables and formal multilevel modeling were not included; therefore, study kebele was used as a proxy indicator for area‐level management and environmental effects.

Although gastrointestinal parasite burden was quantified using FECs (EPG), additional exploratory analyses, in particular, Spearman′s rank correlation analysis, which is appropriate for non‐normally distributed EPG data and ordinal variables such as age and BCS, were not performed due to limitations related to data structure and study design. Future studies are therefore recommended to incorporate correlation‐based analyses to better elucidate the relationship between host characteristics and parasite burden.

## 6. Conclusion and Recommendations

Small ruminant GIT nematode was found to be one of the most important diseases influencing the livestock production in the study areas. In the current study, the overall prevalence of GINs in both sheep and goats was found to be 65.89%. The most prevailing GIT nematode parasites discovered in the current study were *strongyles*, *Strongyloides*, and *Trichuris* species, respectively. The highest prevalence of nematodes was observed in sheep rather than goats. Light infection was the dominant type of infection. Among the considered potential explanatory variables, study sites, species, and BCS had a statistically significant association with the prevalence of nematode parasites. These findings highlight the need for evidence‐based parasite control strategies tailored to pastoral production systems, such as by means of strategic deworming. Moreover, animal health extension workers should work to create abundant awareness in the local community on the methods of control and the impact of gastrointestinal parasites. In addition, the integration of ethnoveterinary practices may offer complementary control options. Edible plant species such as *Ficus* spp., *Punica* spp., and *Trifolium* spp., which possess documented anthelmintic properties, could be incorporated into small ruminant feeding systems to provide partial natural protection against GINs.

## Author Contributions

Yebelayhun Mulugeta conceived and designed the study, developed the research methodology, supervised field activities, performed statistical data analysis, and drafted the manuscript. Getachew Mulugeta contributed to the data collection, supported field supervision, assisted in data interpretation, and critically reviewed and edited the manuscript. Nahom Belay drafted the manuscript, field coordination, and preliminary data organization, and assisted in ensuring data quality. Mahider Tesfaye contributed to data collection, supported field logistics, participated in data organization, and reviewed the manuscript. All authors have made substantial contributions to this work, approved the final version of the manuscript, and agree to be accountable for all aspects of the work.

## Funding

No funding was received for this manuscript.

## Ethics Statement

Ethical approval for this study was obtained from the Institutional Research Ethics Committee of Jinka University, Ethiopia [Approval Number: JKU/RERC/85/2016]. All procedures were conducted in accordance with standard veterinary parasitological guidelines and animal welfare principles. Fecal samples were collected using noninvasive techniques with minimal restraint to avoid stress or harm to animals. Informed verbal consent was obtained from livestock owners prior to sample collection.

## Conflicts of Interest

The authors declare no conflicts of interest.

## Data Availability

The data supporting the findings of this study are available upon request from the corresponding author.
